# Interaction between the *NOS3* Gene and Obesity as a Determinant of Risk of Type 2 Diabetes: The Atherosclerosis Risk in Communities Study

**DOI:** 10.1371/journal.pone.0079466

**Published:** 2013-11-20

**Authors:** Jan Bressler, James S. Pankow, Josef Coresh, Eric Boerwinkle

**Affiliations:** 1 Human Genetics Center, School of Public Health, University of Texas Health Science Center at Houston, Houston, Texas, United States of America; 2 Division of Epidemiology and Community Health, School of Public Health, University of Minnesota, Minneapolis, Minnesota, United States of America; 3 Department of Epidemiology, Johns Hopkins Bloomberg School of Public Health, Baltimore, Maryland, United States of America; 4 Brown Foundation Institute of Molecular Medicine for the Prevention of Human Diseases, University of Texas Health Science Center at Houston, Houston, Texas, United States of America; Vanderbilt University, United States of America

## Abstract

Endothelial nitric oxide synthase 3 (*NOS3*) catalyzes the production of nitric oxide from L-arginine in endothelial cells. Obesity is a modifiable risk factor for diabetes, and obese individuals have been reported to have reduced nitric oxide availability compared to controls whose weight is in the normal range. Since homozygous carriers of the *NOS3* G894T variant are predicted to have decreased enzyme activity, the association between *NOS3* genotype and type 2 diabetes, and possible effect modification by body mass index (BMI) were evaluated. The prevalence of diabetes and BMI was determined at baseline in 14,374 participants 45–66 years of age from the prospective biracial population-based Atherosclerosis Risk in Communities (ARIC) Study of the development of atherosclerosis in four communities in the United States. Individuals with a BMI ≥30 kg/m^2^ were considered obese. Those subjects not meeting the case definition were the comparison groups for the 728 African American and 980 white participants with diabetes. Multivariable logistic regression models adjusted for age, sex, and field center were used to test for main genetic effects and interaction with obesity. Although the *NOS3* G894T variant was not independently associated with diabetes in either African Americans or whites, significant interaction between BMI and the *NOS3* polymorphism indicated that obesity was an effect modifier of diabetes risk for white individuals with the TT genotype (odds ratio (OR) for interaction = 1.65, p = 0.04). In stratified analyses, homozygosity for the *NOS3* T allele in obese white participants but not in those whose BMI <30 kg/m^2^ was associated with an elevated risk of diabetes (OR = 1.47, p = 0.02) when compared to the common GG genotype. These results suggest that interaction between obesity and *NOS3* genotype may be a determinant of diabetes case status in whites in the ARIC cohort. Replication in other populations will be required to confirm these observations.

## Introduction

Diabetes is an important and treatable risk factor for cardiovascular disease. [1] Obesity is an independent risk factor for diabetes and body mass index (BMI) has been commonly used as an index of adiposity. [2] Nitric oxide (NO) is produced by endothelial cells and has been implicated in vascular relaxation in response to multiple agents including acetylcholine, an activity originally attributed to endothelium-derived relaxing factor (EDRF). [3] NO has also been shown to have a role in the regulation of blood pressure and vascular tone [4]. The production of NO from L-arginine is mediated by a constitutive form of NO synthase (eNOS) encoded on chromosome 7q35-36 by the *NOS3* gene. [5] Defects in endothelial cell function and NO production have been described for subjects with atherosclerosis, hypertension, diabetes, as well as obesity.

An association between the *NOS3* G894T (Glu298Asp) single nucleotide polymorphism (SNP) and myocardial infarction, coronary artery disease, stroke, and hypertension has been previously reported although none of these studies included African Americans [6]–[15]. In addition, Monti et al. showed that the 894T allele was more frequent in Italian patients with type 2 diabetes than in normal controls. [16] More recently, an interaction between the G894T SNP and the cholesteryl ester transferase *Taq*I B allele was implicated in an increased risk for diabetes in a case-control study carried out in western Iran. [17]. The presence of the 894T variant has also been associated with the persistence of hyperglycemia over a 5-year period in Chinese subjects with impaired glucose tolerance. [18] When Korean patients with type 2 diabetes were examined, the *NOS3* GT genotype was associated with progression of diabetic nephropathy when compared to the GG genotype. [19] However, an association between the *NOS3* G894T polymorphism and diabetes or diabetes-related traits was not found in all studies. There was no increased risk for diabetes found in Mexican-American participants in the San Antonio Family Diabetes/Gallbladder Study or for type 2 diabetes patients when compared to controls from Japan, Taiwan, or Finland [20]–[23]. The *NOS3* G894T genetic variant was also not associated with albuminuria in European American families, with insulin resistance in subjects with and without diabetes in Japan, with renal disease or diabetic retinopathy in Brazilians with diabetes, or with diabetic retinopathy in a systematic meta-analysis that combined the results of four genetic association studies [24]–[28].

The replacement of guanine by thymine at NOS3 nucleotide 894 results in a change of amino acid from glutamate to aspartate at codon 298 of the mature NOS3 protein. Although this is a conservative amino acid substitution, two groups of investigators have shown that NOS3 with aspartate at position 298 is subject to selective proteolytic cleavage which is predicted to result in absence or reduction of nitric oxide synthase activity in homozygous carriers of the 894T allele. [29], [30] Since obesity has previously been associated with diabetes risk in the Atherosclerosis Risk in Communities (ARIC) Study, and obese individuals have been reported to have reduced NO bioavailability compared to controls whose weight is in the normal range, the aims of this study were to evaluate the association between the *NOS3* G894T polymorphism and prevalent diabetes, and to determine whether interaction between BMI and *NOS3* genotype contributes to susceptibility to the disorder in the large biracial population-based cohort. [31], [32].

## Materials and Methods

### Ethics Statement

All individuals enrolled in the ARIC Study provided written informed consent, and the study design and methods were approved by institutional review boards at the four collaborating medical centers: University of Mississippi Medical Center Institutional Review Board (Jackson Field Center); Wake Forest University Health Sciences Institutional Review Board (Forsyth County Field Center); University of Minnesota Institutional Review Board (Minnesota Field Center); and the Johns Hopkins School of Public Health Institutional Review Board (Washington County Field Center).

### Atherosclerosis Risk in Communities (ARIC) Study

The ARIC Study is a prospective longitudinal investigation of the development of atherosclerosis involving 15,792 individuals aged 45 to 64 years selected by probability sampling from four different communities in the United States. The participants were residents of Forsyth County, NC; Jackson, MS (African Americans only); the northwestern suburbs of Minneapolis, MN; or Washington County, MD at the time of recruitment in 1986–1989. Participants in the ARIC Study were excluded from analysis if they restricted use of their DNA (n = 44), were African American but not from Jackson or Forsyth County (n = 55), were not African American or white (n = 48), or if they were missing *NOS3* genotype (n = 1,161), diabetes case status (n = 70), or BMI (n = 13). When these individuals were excluded, the study sample consisted of 14,401 participants, including 14,374 subjects for whom information concerning diabetes case status at baseline was available. There were 728 African American and 980 white participants with diabetes; the comparison groups were composed of 3,009 and 9,657 individuals, respectively. The initial clinical examination, referred to herein as visit 1, included an interview to collect information concerning the presence of cardiovascular risk factors, socioeconomic status, and family medical history. A detailed description of the ARIC Study has been reported previously. [33].

### Baseline Examination and Laboratory Measures

The prevalence of diabetes was determined at visit 1, and was assessed using a fasting (>8 hours) glucose level that exceeded 7.0 mmol/L, a nonfasting glucose level ≥11.1 mmol/L, and/or a self-reported history of physician diagnosis or use of medication for diabetes. BMI was calculated as weight in kilograms/(height in meters)^2^ from height and weight measurements obtained at the baseline examination. In this study, a BMI ≥25 was used to classify overweight individuals and individuals with a BMI ≥30 were considered obese. [34] Fasting serum glucose was measured by a standard hexokinase method on a Coulter DACOS chemistry analyzer (Coulter Instruments, Fullerton, CA, USA) and the fasting serum insulin level was assessed by radioimmunoassay (^125^Insulin Kit, Cambridge Medical Diagnostics, Billerica, MA, USA). Plasma total cholesterol and triglycerides were measured by enzymatic methods. [35] High density lipoprotein cholesterol (HDL-C) was measured after dextran-magnesium precipitation of non-HDL. [36] Blood pressure was measured three times while seated using a random-zero sphygmomanometer and the last two measurements were averaged for analysis. Information pertaining to either maternal or paternal history of diabetes was obtained during interviews carried out during the first clinical examination.

### Genotype Determination

Genotyping of the *NOS3* G894T polymorphism (rs1799983) was performed using the TaqMan assay (Applied Biosystems, Foster City, CA, USA). Oligonucleotide sequences for polymerase chain reaction (PCR) primers and TaqMan probes are available upon request from the authors. Allele detection was performed using the ABI Prism 7700 Sequence Detection System (Applied Biosystems). The genotype call rate, or the percentage of samples to which a genotype was assigned, was 93.0% and was determined prior to exclusion of individuals from the analysis. The genotyping success rate was also assessed by analyzing the concordance between genotypes for 680 pairs of blind duplicates included with the DNA samples from the study participants. The simple kappa coefficient, an index of the percent agreement between measurements corrected for agreement occurring by chance, was 0.91. [37].

### Statistical Analysis

All statistical analyses were carried out using the Stata statistical software programs (StataCorp, College Station, TX, USA). The hypothesis that observed genotypes were in Hardy-Weinberg equilibrium was tested using a χ^2^ goodness-of-fit test. The proportions, means and standard deviations (SD) were calculated for established risk factors for diabetes for both the prevalent diabetes cases and for the comparison groups of control individuals who did not meet the case definition. Multivariable logistic regression was used to evaluate the relationship between diabetes case status, *NOS3* G894T genotype, and measures of obesity under a general genotypic (two-degree of freedom) genetic model. Prevalence odds ratios were adjusted for age, gender, and field center. Interaction between BMI and *NOS3* was analyzed in regression models adjusted for the same covariates used to assess the main effects of obesity and the *NOS3* G894T polymorphism on diabetes risk, and that also included interaction terms for genotype by obesity. A two-sided p-value <0.05 was considered statistically significant for all tests. The results of all of the statistical analyses are reported separately by self-reported racial group. Power analyses for the main effect of the *NOS3* G894T variant on diabetes case status were carried out with the program Quanto using a p-value of 0.05, and the sample size, allele frequencies, and prevalence of diabetes for each racial group. The power to detect interaction between obesity and the *NOS3* G894 TT genotype was evaluated by also including the prevalence of obesity, and the genetic odds ratio and environmental odds ratio for association with diabetes observed in the study for each race. [38].

## Results

The means, standard deviations, and proportions for each of the diabetes risk factors are shown for the study sample stratified by diabetes case status and race in Table 1. The clinical and demographic characteristics differed significantly between individuals with diabetes and participants without diabetes for both whites and African Americans. Prevalent diabetes cases were older, and more were obese or overweight compared to non-cases. There was also a significantly greater frequency of males and fewer females in the group of white subjects with diabetes when compared to the non-case group (p = 7.05×10^−5^), while the proportion of persons of each gender was not significantly related to case status for African Americans (p = 0.118) in the study sample.

**Table 1:** Clinical and demographic characteristics stratified by race and diabetes case status.

	White			African American		
Characteristics	Diabetes	Non-Cases	p	Diabetes	Non-Cases	P
Number	980	9,657		728	3,009	
Age, years	56.2 (5.6)^a^	54.2 (5.7)^a^	<1.0×10^−6^	55.3 (5.7)^a^	53.2 (5.8)^a^	<1.0×10^−6^
Glucose, mmol/L	9.7(3.9)^a^	5.5 (0.5)^a^	<1.0×10^−6^	11.1 (5.0)a,g	5.5 (0.6)^a^	<1.0×10^−6^
Insulin, pmol/L	225.2 (438.3)a,b	72.9 (54.7)^a^	<1.0×10^−6^	339.3 (631.6)a,h	94.6 (69.9)^a^	<1.0×10^−6^
DBP, mm Hg	72.9 (11.0)a,b	71.3 (10.0)a,d	3.08×10^−6^	78.5 (11.9)^a^	80.1 (12.3)a,b	1.35×10^−3^
SBP, mm Hg	127.0 (18.4)a,b	117.7 (16.7)a,e	<1.0×10^−6^	133.7 (22.6)^a^	127.5 (20.6)a,b	<1.0×10^−6^
HDL, mmol/L	1.06 (0.35)a,c	1.33 (0.43)a,f	<1.0×10^−6^	1.27 (0.38)a,i	1.46 (0.46)a,k	<1.0×10^−6^
Triglycerides, mmol/L	2.40 (1.78)a,c	1.47 (0.89)a,f	<1.0×10^−6^	1.72 (1.30)a,j	1.19 (0.77)a,k	<1.0×10^−6^
Male	524 (53.5)^l^	4,521 (46.8)^l^	7.05×10^−5^ *	260 (35.7)^l^	1,169 (38.8)^l^	1.18×10^−1^ *
BMI ≥30 kg/m^2^	462 (47.1)^l^	1,960 (20.3)^l^	<1.0×10^−6^ *	420 (57.7)^l^	1,081 (35.9)^l^	<1.0×10^−6^ *
BMI ≥25 kg/m^2^	829 (84.6)^l^	5,841 (60.5)^l^	<1.0×10^−6^ *	652 (89.6)^l^	2,265 (75.3)^l^	<1.0×10^−6^ *
Family history diabetes	487 (49.7)^l^	2,709 (28.0)^l^	<1.0×10^−6^ *	432 (59.3)^l^	1.310 (43.5)^l^	<1.0×10^−6^ *

p, p-value, significance of difference between group means determined by t-test; DBP, diastolic blood pressure; SBP, systolic blood pressure; HDL, high density lipoprotein; BMI, body mass index;

a mean and standard deviation;

b missing values (n = 1);

c missing values (n = 2);

d missing values (n = 5);

e missing values (n = 4);

f missing values (n = 12);

g missing values (n = 15);

h missing values (n = 16);

i missing values (n = 21);

j missing values (n = 20);

k missing values (n = 55);

l number and percentage;

* p-value Pearson’s chi-squared test.

The results of genotyping the *NOS3* G894T SNP are presented in Table 2. The allele and genotype frequencies for the *NOS3* polymorphism were in accordance with Hardy-Weinberg equilibrium expectations for both African American (p = 0.62) and white (p = 0.95) individuals in the study, and are consistent with those previously reported for the Yoruba in Ibadan, Nigeria and CEPH (Utah residents with ancestry from northern and western Europe) populations included in the International HapMap Project, [39] and in a study of candidate genes associated with childhood obesity. [40] The proportions of each of the three genotypes did not differ significantly between cases and non-cases for either racial group. Since the genotype frequencies for the *NOS3* G894T polymorphism were significantly different when African American and white individuals in the study were compared (p = <1.0×10^−6^), the results of the statistical analyses are reported separately by race.

**Table 2:** *NOS3* G894T genotype and allele frequencies stratified by race and diabetes case status.

		White					African American				
SNP	Genotype/Allele	Diabetes		Non-Cases		p^1^	Diabetes		Non-Cases		p^1^
		n	%	n	%		n	%	n	%	
G894T	GG	450	45.9	4,506	46.7	0.82	580	79.7	2,338	77.7	0.50
	GT	426	43.5	4,181	43.3		139	19.1	626	20.8	
	TT	104	10.6	970	10.0		9	1.2	45	1.5	
	G	1,326	67.6	13,193	68.3		1,299	89.2	5,302	88.1	
	T	634	32.4	6,121	31.7		157	10.8	716	11.9	

SNP, single nucleotide polymorphism; p^1^, p-value Pearson’s chi-squared test for comparison of proportions of *NOS3* G894T genotypes; n, number.

Multivariable logistic regression models were used to examine the association of sequence variation in the *NOS3* gene and diabetes case status. Analysis of the main effect of the *NOS3* G894T polymorphism on diabetes risk after adjustment for age, sex, and field center showed no significant association when either the group of white participants or African American participants was evaluated as a whole (Tables 3 and 4). Obesity as assessed by a BMI ≥30 kg/m^2^ was significantly associated with diabetes prevalence in both white and African American individuals. In white but not in African-American study participants, the relationship between the G894T polymorphism and diabetes risk was modified by BMI with evidence for interaction between obesity and the TT genotype (OR for interaction = 1.65, p = 0.04) (Tables 3 and 4). In subsequent analyses stratified by levels of BMI, homozygosity for the *NOS3* 894T allele in white obese individuals was shown to be significantly associated with an increased risk for diabetes when compared to the most common GG genotype (OR = 1.47, p = 0.02), while no variation in susceptibility with genotype was found for white individuals whose BMI <30 kg/m^2^ (Table 3). In secondary analyses, application of the same logistic models showed that overweight white individuals homozygous for the *NOS3* 894T allele did not incur a significant risk of diabetes when compared to carriers of two G alleles, and there was no association between the TT genotype and susceptibility to diabetes in those whose BMI was <25 kg/m^2^ (data not shown).

**Table 3:** Association of *NOS3* genotype and diabetes case status in whites stratified by obesity.

Variable	Genotype	BMI	kg/m^2^	n	OR	95% CI	p
			All	10,637			
Obesity(BMI ≥30 kg/m^2^)					3.54	3.08–4.06	<0.001
Interaction^1^					1.65	1.03–2.64	0.04
G894T	GG				**	**	**
	GT				1.02	0.89–1.18	0.73
	TT				1.07	0.86–1.35	0.54
			<30	8,215			
G894T	GG				**	**	**
	GT				1.02	0.84–1.23	0.86
	TT				0.90	0.65–1.24	0.50
			≥30	2,422			
G894T	GG				**	**	**
	GT				1.06	0.85–1.32	0.60
	TT				1.47	1.05–2.07	0.02

*NOS3*, endothelial nitric oxide synthase 3; n, number; OR, odds ratio; CI, confidence interval; p, p-value adjusted for age, sex, and field center; BMI, body mass index,

1 interaction term for *NOS3* G894 TT genotype and obesity;

** reference genotype.

**Table 4:** Association of *NOS3* genotype and diabetes case status in African Americans stratified by obesity.

Variable	Genotype	BMI	kg/m^2^	n	OR	95% CI	p
			All	3,737			
Obesity(BMI ≥30 kg/m^2^)					2.53	2.13–3.01	<0.001
Interaction^1^					0.47	0.11–2.06	0.32
G894T	GG				**	**	**
	GT				0.91	0.74–1.12	0.37
	TT				0.79	0.38–1.63	0.52
			<30	2,236			
G894T	GG				**	**	**
	GT				0.97	0.71–1.31	0.83
	TT				1.12	0.42–3.00	0.82
			≥30	1,501			
G894T	GG				**	**	**
	GT				0.86	0.64–1.15	0.30
	TT				0.53	0.18–1.58	0.25

*NOS3*, endothelial nitric oxide synthase 3; n, number; OR, odds ratio; CI, confidence interval; p, p-value adjusted for age, sex, and field center; BMI, body mass index,

1 interaction term for *NOS3* G894 TT genotype and obesity;

** reference genotype.

In contrast to the results described above, the effect of *NOS3* G894T genotype on diabetes case status did not appear to vary with level of BMI in African American participants (Table 4) although there are only a small number of individuals with the TT genotype reflecting the low minor allele frequency (Table 2).

## Discussion

Endothelial dysfunction, characterized by impaired vasodilation and availability of NO, is a common feature of both diabetes and obesity [32], [41]–[44]. Obesity is a risk factor for diabetes, and is associated with the development of insulin resistance in peripheral tissues. [45] Changes in levels of several protein mediators released by excess adipose tissue including tumor necrosis factor, interleukin 6 (interferon, beta 2), resistin, leptin, and adiponectin as well as free fatty acids can result in impaired insulin action in liver and skeletal muscle [46]–[50]. Both insulin resistance and endothelial dysfunction have been shown to precede the abnormal glucose levels characteristic of diabetes and are already present in individuals at known risk for the disease such as those with a positive family history. [32], [45], [51], [52] Insulin has direct effects on the vasculature by stimulating the production of NO in endothelial cells through the P13-kinase/Akt pathway [53]–[57]. This capacity is diminished in insulin-resistant individuals despite increased production of insulin by the pancreas leading to decreased vascular reactivity. Superoxide produced in the vascular wall may further decrease the bioavailability of NO and enhance oxidative stress through the generation of peroxynitrite and increased synthesis of reactive oxygen species due to NOS3 uncoupling [58]. Conversely, weight loss leads to improved endothelial function and insulin sensitivity [59]–[63]. It is therefore possible to speculate that in obese subjects, genetic polymorphisms such as *NOS3* G894T that influence the basal level of NO production contribute to progression to diabetes under conditions where the amount may be further reduced as a consequence of insulin resistance, whereas this relationship is not seen in individuals who are overweight or whose weight is in the normal range.

Interestingly, mice with a heterozygous deletion of *Nos3* fed a standard diet had normal insulin sensitivity, while *Nos3* ^+/−^ mice fed a high fat diet for eight weeks showed fasting hyperinsulinemia and reduced insulin-stimulated glucose utilization, possibly indicating a critical role for environmental factors in metabolic disease. [64] In this context, variation in other genetic or environmental factors such as nutrition that may contribute to the prevalence of type 2 diabetes in the two racial groups, as well as the significant difference in allele frequencies for the G894T variant, may at least partially account for the disparity in the association between the *NOS3* SNP and diabetes risk in obese subjects when white and African American participants were compared. There was also lack of statistical power to detect an effect size for interaction between the TT genotype and obesity comparable to that found in whites. While there was 70% power to detect an OR of 1.70 for interaction for whites under a recessive genetic model, there was 10% power to observe the same OR for African Americans.

Monti et al. have previously reported that the frequency of the *NOS3* 894T allele was higher in a sample of 159 patients with diabetes than in 207 healthy control subjects, and a significantly elevated waist-to-hip ratio was observed in the individuals with type 2 diabetes who were homozygous TT carriers. [16] In a longitudinal study of the development of cardiovascular risk factors from childhood to adulthood, African- and European-American carriers of the *NOS3* 894T allele were reported to have a higher mean BMI, waist circumference, and sum of skinfolds if they were of low socioeconomic status. [40] The results of studies in laboratory animals also support a role for *NOS3* in the determination of body size. Mice deficient in *Nos3* due to introduction of a targeted deletion into the mouse genome were shown to have reduced body weight that was more prominent in females. However, there was no evidence of statistical interaction between gender and genotype in animals carrying the mutation. [65].

Although an important limitation of this study is that the measurement of BMI in prevalent diabetes cases may not reflect adiposity at the time of disease onset, the results reported here may explain the failure to date to detect *NOS3* genetic variants in genome-wide association studies of diabetes that did not explicitly include interaction between genes and the environment in the study design. While there may be no apparent effect of the *NOS3* G894T variant when a particular population is examined as a whole, it may be possible that the influence of the polymorphism on diabetes risk can only be observed in well-defined subgroups of obese individuals, although replication in other cohorts will be required to support this speculation. As in any cross-sectional observational study of a single candidate SNP, another caveat when interpreting these results is that the *NOS3* G894T variant may participate in other gene-gene or gene-environment interactions besides that hypothesized for obesity so that its total effect on diabetes susceptibility may not have been addressed. Identification of a significant gene-environment interaction could also be dependent on the case definition used to classify diabetes cases as well as the comparison group of non-cases. In an effort to explore the impact of including individuals with prediabetes as part of the control group, persons without diabetes as defined in the ARIC Study but who had measurements of fasting glucose between 5.54 mmol/L and 7.00 mmol/L were excluded from the analyses (N = 5,350; African American = 1,294; white = 4,056). When this was done, the OR for the interaction term for obesity and the *NOS3* G894T TT genotype in whites was 1.71 (95% confidence interval (CI) = 1.02–2.87) and the p-value for interaction was 0.042, only slightly changed from the values found when these study subjects were included (Table 3). This result suggests that detection of the marginally significant interaction between *NOS3* genotype and obesity was not due to bias towards the null attributable to inclusion of this subgroup. In addition, further adjustment of the regression models for family history as a proxy for shared genetic and environmental factors did not attenuate the association (OR = 1.84; 95% CI = 1.14–2.95; p-value for interaction = 0.012) in secondary analyses.

The strengths of the study include the direct measurement of BMI as the environmental exposure, and the large well-phenotyped study population with adequate power to detect an interaction in whites of the magnitude previously reported in analyses of modulation of the effect of established genetic risk factors for type 2 diabetes by lifestyle differences. For example, an interaction between dietary carbohydrate and a transcription factor 7-like 2 (*TCF7L2*) polymorphism (p = 0.03) was observed in the Nurses’ Health Study where there was greater susceptibility seen in women with the highest glycemic load (OR = 1.62; 95% CI = 1.32–2.00) for each addition of the rs115537 T allele compared to those with the lowest glycemic load (OR = 1.15; 95% CI = 0.92–1.45). [66], [67] The risk of diabetes for individuals who consumed a high fat diet was also recently shown to vary by genotype at the peroxisome proliferator-activated receptor gamma (*PPARG*) locus. The adverse effects of elevated fat intake were more prominent for Pro12 homozygotes than for Ala12 carriers (OR = 1.73; 95% CI = 1.19–2.52; p = 0.004) in participants in the D.E.S.I.R. cohort study genotyped for the Pro12Ala variant (p-value for interaction = 0.05) [68]–[70]. Similarly, in a study of effect modification by physical activity of the association between 17 SNPs identified in genome-wide association studies of diabetes and incident disease among 16,003 Swedish subjects, the corrected p-value for interaction was 0.0068 for rs4430796 located within the gene encoding the transcription factor HNF1 homeobox B (HNF1B). The protection conferred by physical activity was found to be decreased in carriers of the minor A allele so that there was a higher risk of diabetes in active individuals (hazard rate ratio (HRR) = 1.10; 95% CI = 0.74–0.96; p = 0.007) than in sedentary individuals (HRR = 0.85; 95% CI = 1.03–1.18; p = 0.011). [71], [72].

Assuming the interaction between a common *NOS3* sequence variant and obesity reported here is only a single occurrence of a more general phenomenon, large well-powered studies carried out in populations where phenotypic heterogeneity has been minimized may be required to bridge the gap between the substantial heritability estimated for many complex disorders and the proportion of interindividual variation thus far accounted for by common sequence differences. [73].

## References

[1] Haffner SM, Lehto S, Ronnemaa T, Pyorala K, Laakso M (1998) Mortality from coronary heart disease in subjects with type 2 diabetes and in nondiabetic subjects with and without prior myocardial infarction. N Engl J Med 339: 229–234. 9673301 10.1056/NEJM199807233390404

[2] Stern MP, Haffner SM (1986) Body fat distribution and hyperinsulinemia as risk factors for diabetes and cardiovascular disease. Arteriosclerosis 6: 123–130. 3513749 10.1161/01.atv.6.2.123

[3] Furchgott RF, Zawadzki JV (1980) The obligatory role of endothelial cells in the relaxation of arterial smooth muscle by acetylcholine. Nature 288: 373–376. 6253831 10.1038/288373a0

[4] Rees DD, Palmer RM, Moncada S (1989) Role of endothelium-derived nitric oxide in the regulation of blood pressure. Proc Natl Acad Sci U S A 86: 3375–3378. 2497467 10.1073/pnas.86.9.3375 PMC287135

[5] Marsden PA, Heng HH, Scherer SW, Stewart RJ, Hall AV, et al (1993) Structure and chromosomal localization of the human constitutive endothelial nitric oxide synthase gene. J Biol Chem 268: 17478–17488. 7688726

[6] Yoshimura M, Yasue H, Nakayama M, Shimasaki Y, Sumida H, et al (1998) A missense Glu298Asp variant in the endothelial nitric oxide synthase gene is associated with coronary spasm in the Japanese. Hum Genet 103: 65–69. 9737779 10.1007/s004390050785

[7] Hibi K, Ishigami T, Tamura K, Mizushima S, Nyui N, et al (1998) Endothelial nitric oxide synthase gene polymorphism and acute myocardial infarction. Hypertension 32: 521–526. 9740620 10.1161/01.hyp.32.3.521

[8] Casas JP, Bautista LE, Humphries SE, Hingorani AD (2004) Endothelial nitric oxide synthase genotype and ischemic heart disease: meta-analysis of 26 studies involving 23028 subjects. Circulation 109: 1359–1365. 15007011 10.1161/01.CIR.0000121357.76910.A3

[9] Shimasaki Y, Yasue H, Yoshimura M, Nakayama M, Kugiyama K, et al (1998) Association of the missense Glu298Asp variant of the endothelial nitric oxide synthase gene with myocardial infarction. J Am Coll Cardiol 31: 1506–1510. 9626827 10.1016/s0735-1097(98)00167-3

[10] Hingorani AD, Liang CF, Fatibene J, Lyon A, Monteith S, et al (1999) A common variant of the endothelial nitric oxide synthase (Glu298–>Asp) is a major risk factor for coronary artery disease in the UK. Circulation 100: 1515–1520. 10510054 10.1161/01.cir.100.14.1515

[11] Casas JP, Cavalleri GL, Bautista LE, Smeeth L, Humphries SE, et al (2006) Endothelial nitric oxide synthase gene polymorphisms and cardiovascular disease: a HuGE review. Am J Epidemiol 164: 921–935. 17018701 10.1093/aje/kwj302

[12] Berger K, Stogbauer F, Stoll M, Wellmann J, Huge A, et al (2007) The glu298asp polymorphism in the nitric oxide synthase 3 gene is associated with the risk of ischemic stroke in two large independent case-control studies. Hum Genet 121: 169–178. 17165044 10.1007/s00439-006-0302-2

[13] Miyamoto Y, Saito Y, Kajiyama N, Yoshimura M, Shimasaki Y, et al (1998) Endothelial nitric oxide synthase gene is positively associated with essential hypertension. Hypertension 32: 3–8. 9674630 10.1161/01.hyp.32.1.3

[14] Pereira TV, Rudnicki M, Cheung BM, Baum L, Yamada Y, et al (2007) Three endothelial nitric oxide (NOS3) gene polymorphisms in hypertensive and normotensive individuals: meta-analysis of 53 studies reveals evidence of publication bias. J Hypertens 25: 1763–1774. 17762636 10.1097/HJH.0b013e3281de740d

[15] Misono M, Maeda S, Iemitsu M, Nakata Y, Otsuki T, et al (2009) Combination of polymorphisms in the beta2-adrenergic receptor and nitric oxide synthase 3 genes increases the risk for hypertension. J Hypertens 27: 1377–1383. 19373110 10.1097/HJH.0b013e32832b7ead

[16] Monti LD, Barlassina C, Citterio L, Galluccio E, Berzuini C, et al (2003) Endothelial nitric oxide synthase polymorphisms are associated with type 2 diabetes and the insulin resistance syndrome. Diabetes 52: 1270–1275. 12716763 10.2337/diabetes.52.5.1270

[17] Rahimi Z, Nourozi-Rad R, Rahimi Z, Parsian A (2012) Strong interaction between T allele of endothelial nitric oxide synthase with B1 allele of cholesteryl ester transfer protein TaqIB highly elevates the risk of coronary artery disease and type 2 diabetes mellitus. Hum Genomics 6: 20. 23157875 10.1186/1479-7364-6-20 PMC3500247

[18] Tso AW, Tan KC, Wat NM, Janus ED, Lam TH, et al (2006) Endothelial nitric oxide synthase G894T (Glu298Asp) polymorphism was predictive of glycemic status in a 5-year prospective study of Chinese subjects with impaired glucose tolerance. Metabolism 55: 1155–1158. 16919532 10.1016/j.metabol.2006.04.012

[19] Shin Shin Y, Baek SH, Chang KY, Park CW, Yang CW, et al (2004) Relations between eNOS Glu298Asp polymorphism and progression of diabetic nephropathy. Diabetes Res Clin Pract 65: 257–265. 15331206 10.1016/j.diabres.2004.01.010

[20] Thameem F, Puppala S, Arar NH, Stern MP, Blangero J, et al (2008) Endothelial nitric oxide synthase (eNOS) gene polymorphisms and their association with type 2 diabetes-related traits in Mexican Americans. Diab Vasc Dis Res 5: 109–113. 18537098 10.3132/dvdr.2008.018 PMC3336872

[21] Awata T, Neda T, Iizuka H, Kurihara S, Ohkubo T, et al (2004) Endothelial nitric oxide synthase gene is associated with diabetic macular edema in type 2 diabetes. Diabetes Care 27: 2184–2190. 15333482 10.2337/diacare.27.9.2184

[22] Hu CJ, Wang CH, Lee JH, Hsieh CM, Cheng CC, et al (2007) Association between polymorphisms of ACE, B2AR, ANP and ENOS and cardiovascular diseases: a community-based study in the Matsu area. Clin Chem Lab Med 45: 20–25. 17243909 10.1515/CCLM.2007.014

[23] Ukkola O, Erkkila PH, Savolainen MJ, Kesaniemi YA (2001) Lack of association between polymorphisms of catalase, copper-zinc superoxide dismutase (SOD), extracellular SOD and endothelial nitric oxide synthase genes and macroangiopathy in patients with type 2 diabetes mellitus. J Intern Med 249: 451–459. 11350569 10.1046/j.1365-2796.2001.00828.x

[24] Liu Y, Burdon KP, Langefeld CD, Beck SR, Wagenknecht LE, et al (2005) T-786C polymorphism of the endothelial nitric oxide synthase gene is associated with albuminuria in the diabetes heart study. J Am Soc Nephrol 16: 1085–1090. 15743995 10.1681/ASN.2004100817

[25] Ohtoshi K, Yamasaki Y, Gorogawa S, Hayaishi-Okano R, Node K, et al (2002) Association of (-)786T-C mutation of endothelial nitric oxide synthase gene with insulin resistance. Diabetologia 45: 1594–1601. 12436344 10.1007/s00125-002-0922-6

[26] Santos KG, Crispim D, Canani LH, Ferrugem PT, Gross JL, et al (2011) Association of eNOS gene polymorphisms with renal disease in Caucasians with type 2 diabetes. Diabetes Res Clin Pract 91: 353–362. 21255858 10.1016/j.diabres.2010.12.029

[27] Santos KG, Crispim D, Canani LH, Ferrugem PT, Gross JL, et al (2012) Relationship of endothelial nitric oxide synthase (eNOS) gene polymorphisms with diabetic retinopathy in Caucasians with type 2 diabetes. Ophthalmic Genet 33: 23–27. 22017289 10.3109/13816810.2011.620057

[28] Abhary S, Hewitt AW, Burdon KP, Craig JE (2009) A systematic meta-analysis of genetic association studies for diabetic retinopathy. Diabetes 58: 2137–2147. 19587357 10.2337/db09-0059 PMC2731535

[29] Tesauro M, Thompson WC, Rogliani P, Qi L, Chaudhary PP, et al (2000) Intracellular processing of endothelial nitric oxide synthase isoforms associated with differences in severity of cardiopulmonary diseases: cleavage of proteins with aspartate vs. glutamate at position 298. Proc Natl Acad Sci U S A 97: 2832–2835. 10717002 10.1073/pnas.97.6.2832 PMC16015

[30] Persu A, Stoenoiu MS, Messiaen T, Davila S, Robino C, et al (2002) Modifier effect of ENOS in autosomal dominant polycystic kidney disease. Hum Mol Genet 11: 229–241. 11823442 10.1093/hmg/11.3.229

[31] Bang H, Edwards AM, Bomback AS, Ballantyne CM, Brillon D, et al (2009) Development and validation of a patient self-assessment score for diabetes risk. Ann Intern Med 151: 775–783. 19949143 10.1059/0003-4819-151-11-200912010-00005 PMC3633111

[32] Steinberg HO, Chaker H, Leaming R, Johnson A, Brechtel G, et al (1996) Obesity/insulin resistance is associated with endothelial dysfunction. Implications for the syndrome of insulin resistance. J Clin Invest 97: 2601–2610. 8647954 10.1172/JCI118709 PMC507347

[33] The ARIC investigators (1989) The Atherosclerosis Risk in Communities (ARIC) Study: design and objectives. Am J Epidemiol 129: 687–702. 2646917

[34] Obesity: preventing and managing the global epidemic. (2000) Report of a WHO consultation. World Health Organ Tech Rep Ser 894: i–xii, 1–253. 11234459

[35] Siedel J, Hagele EO, Ziegenhorn J, Wahlefeld AW (1983) Reagent for the enzymatic determination of serum total cholesterol with improved lipolytic efficiency. Clin Chem 29: 1075–1080. 6851096

[36] Warnick GR, Benderson J, Albers JJ (1982) Dextran sulfate-Mg2+ precipitation procedure for quantitation of high-density-lipoprotein cholesterol. Clin Chem 28: 1379–1388. 7074948

[37] Cohen J (1960) A coefficient of agreement for nominal scales. Educational and Psychological Measurement 20: 37–46.

[38] Gauderman W, Morrison J (2006) QUANTO 1.1: A computer program for power and sample size calculations for genetic epidemiology studies. Available: http://hydra.usc.edu/gxe/Accessed 2013 October 15.

[39] International HapMap Consortium, Frazer KA, Ballinger DG, Cox DR, Hinds DA, et al (2007) A second generation human haplotype map of over 3.1 million SNPs. Nature 449: 851–861. 17943122 10.1038/nature06258 PMC2689609

[40] Podolsky RH, Barbeau P, Kang HS, Zhu H, Treiber FA, et al (2007) Candidate genes and growth curves for adiposity in African- and European-American youth. Int J Obes (Lond) 31: 1491–1499. 17621313 10.1038/sj.ijo.0803673

[41] Hink U, Li H, Mollnau H, Oelze M, Matheis E, et al (2001) Mechanisms underlying endothelial dysfunction in diabetes mellitus. Circ Res 88: E14–22. 11157681 10.1161/01.res.88.2.e14

[42] Johnstone MT, Creager SJ, Scales KM, Cusco JA, Lee BK, et al (1993) Impaired endothelium-dependent vasodilation in patients with insulin-dependent diabetes mellitus. Circulation 88: 2510–2516. 8080489 10.1161/01.cir.88.6.2510

[43] De Vriese AS, Verbeuren TJ, Van de Voorde J, Lameire NH, Vanhoutte PM (2000) Endothelial dysfunction in diabetes. Br J Pharmacol 130: 963–974. 10882379 10.1038/sj.bjp.0703393 PMC1572156

[44] McVeigh GE, Brennan GM, Johnston GD, McDermott BJ, McGrath LT, et al (1992) Impaired endothelium-dependent and independent vasodilation in patients with type 2 (non-insulin-dependent) diabetes mellitus. Diabetologia 35: 771–776. 1511805 10.1007/BF00429099

[45] Kahn BB, Flier JS (2000) Obesity and insulin resistance. J Clin Invest 106: 473–481. 10953022 10.1172/JCI10842 PMC380258

[46] Caballero AE (2003) Endothelial dysfunction in obesity and insulin resistance: a road to diabetes and heart disease. Obes Res 11: 1278–1289. 14627747 10.1038/oby.2003.174

[47] Rask-Madsen C, King GL (2007) Mechanisms of Disease: endothelial dysfunction in insulin resistance and diabetes. Nat Clin Pract Endocrinol Metab 3: 46–56. 17179929 10.1038/ncpendmet0366

[48] Kershaw EE, Flier JS (2004) Adipose tissue as an endocrine organ. J Clin Endocrinol Metab 89: 2548–2556. 15181022 10.1210/jc.2004-0395

[49] Meier U, Gressner AM (2004) Endocrine regulation of energy metabolism: review of pathobiochemical and clinical chemical aspects of leptin, ghrelin, adiponectin, and resistin. Clin Chem 50: 1511–1525. 15265818 10.1373/clinchem.2004.032482

[50] Huang PL (2009) eNOS, metabolic syndrome and cardiovascular disease. Trends Endocrinol Metab 20: 295–302. 19647446 10.1016/j.tem.2009.03.005 PMC2731551

[51] Haffner SM (2003) Pre-diabetes, insulin resistance, inflammation and CVD risk. Diabetes Res Clin Pract 61 Suppl 1S9–S18. 12880690 10.1016/s0168-8227(03)00122-0

[52] Caballero AE, Arora S, Saouaf R, Lim SC, Smakowski P, et al (1999) Microvascular and macrovascular reactivity is reduced in subjects at risk for type 2 diabetes. Diabetes 48: 1856–1862. 10480619 10.2337/diabetes.48.9.1856

[53] Kuboki K, Jiang ZY, Takahara N, Ha SW, Igarashi M, et al (2000) Regulation of endothelial constitutive nitric oxide synthase gene expression in endothelial cells and in vivo : a specific vascular action of insulin. Circulation 101: 676–681. 10673261 10.1161/01.cir.101.6.676

[54] Montagnani M, Chen H, Barr VA, Quon MJ (2001) Insulin-stimulated activation of eNOS is independent of Ca2+ but requires phosphorylation by Akt at Ser(1179). J Biol Chem 276: 30392–30398. 11402048 10.1074/jbc.M103702200

[55] Montagnani M, Ravichandran LV, Chen H, Esposito DL, Quon MJ (2002) Insulin receptor substrate-1 and phosphoinositide-dependent kinase-1 are required for insulin-stimulated production of nitric oxide in endothelial cells. Mol Endocrinol 16: 1931–1942. 12145346 10.1210/me.2002-0074

[56] Fleming I, Busse R (1999) Signal transduction of eNOS activation. Cardiovasc Res 43: 532–541. 10690325 10.1016/s0008-6363(99)00094-2

[57] Zeng G, Nystrom FH, Ravichandran LV, Cong LN, Kirby M, et al (2000) Roles for insulin receptor, PI3-kinase, and Akt in insulin-signaling pathways related to production of nitric oxide in human vascular endothelial cells. Circulation 101: 1539–1545. 10747347 10.1161/01.cir.101.13.1539

[58] Zou MH, Cohen R, Ullrich V (2004) Peroxynitrite and vascular endothelial dysfunction in diabetes mellitus. Endothelium 11: 89–97. 15370068 10.1080/10623320490482619

[59] Balkestein EJ, van Aggel-Leijssen DP, van Baak MA, Struijker-Boudier HA, Van Bortel LM (1999) The effect of weight loss with or without exercise training on large artery compliance in healthy obese men. J Hypertens 17: 1831–1835. 10703876 10.1097/00004872-199917121-00008

[60] Ziccardi P, Nappo F, Giugliano G, Esposito K, Marfella R, et al (2002) Reduction of inflammatory cytokine concentrations and improvement of endothelial functions in obese women after weight loss over one year. Circulation 105: 804–809. 11854119 10.1161/hc0702.104279

[61] Hamdy O, Ledbury S, Mullooly C, Jarema C, Porter S, et al (2003) Lifestyle modification improves endothelial function in obese subjects with the insulin resistance syndrome. Diabetes Care 26: 2119–2125. 12832323 10.2337/diacare.26.7.2119

[62] Sciacqua A, Candigliota M, Ceravolo R, Scozzafava A, Sinopoli F, et al (2003) Weight loss in combination with physical activity improves endothelial dysfunction in human obesity. Diabetes Care 26: 1673–1678. 12766092 10.2337/diacare.26.6.1673

[63] Rittig K, Hieronimus A, Thamer C, Machann J, Peter A, et al (2010) Reducing visceral adipose tissue mass is essential for improving endothelial function in type 2 diabetes prone individuals. Atherosclerosis 212: 575–579. 20667538 10.1016/j.atherosclerosis.2010.06.042

[64] Cook S, Hugli O, Egli M, Menard B, Thalmann S, et al (2004) Partial gene deletion of endothelial nitric oxide synthase predisposes to exaggerated high-fat diet-induced insulin resistance and arterial hypertension. Diabetes 53: 2067–2072. 15277387 10.2337/diabetes.53.8.2067

[65] Shesely EG, Maeda N, Kim HS, Desai KM, Krege JH, et al (1996) Elevated blood pressures in mice lacking endothelial nitric oxide synthase. Proc Natl Acad Sci U S A 93: 13176–13181. 8917564 10.1073/pnas.93.23.13176 PMC24066

[66] Grant SF, Thorleifsson G, Reynisdottir I, Benediktsson R, Manolescu A, et al (2006) Variant of transcription factor 7-like 2 (TCF7L2) gene confers risk of type 2 diabetes. Nat Genet 38: 320–323. 16415884 10.1038/ng1732

[67] Cornelis MC, Qi L, Kraft P, Hu FB (2009) TCF7L2, dietary carbohydrate, and risk of type 2 diabetes in US women. Am J Clin Nutr 89: 1256–1262. 19211816 10.3945/ajcn.2008.27058 PMC2667467

[68] Deeb SS, Fajas L, Nemoto M, Pihlajamaki J, Mykkanen L, et al (1998) A Pro12Ala substitution in PPARgamma2 associated with decreased receptor activity, lower body mass index and improved insulin sensitivity. Nat Genet 20: 284–287. 9806549 10.1038/3099

[69] Memisoglu A, Hu FB, Hankinson SE, Liu S, Meigs JB, et al (2003) Prospective study of the association between the proline to alanine codon 12 polymorphism in the PPARgamma gene and type 2 diabetes. Diabetes Care 26: 2915–2917. 14514601 10.2337/diacare.26.10.2915

[70] Lamri A, Abi Khalil C, Jaziri R, Velho G, Lantieri O, et al (2012) Dietary fat intake and polymorphisms at the PPARG locus modulate BMI and type 2 diabetes risk in the D.E.S.I.R. prospective study. Int J Obes (Lond) 36: 218–224. 21540831 10.1038/ijo.2011.91

[71] Gudmundsson J, Sulem P, Steinthorsdottir V, Bergthorsson JT, Thorleifsson G, et al (2007) Two variants on chromosome 17 confer prostate cancer risk, and the one in TCF2 protects against type 2 diabetes. Nat Genet 39: 977–983. 17603485 10.1038/ng2062

[72] Brito EC, Lyssenko V, Renstrom F, Berglund G, Nilsson PM, et al (2009) Previously associated type 2 diabetes variants may interact with physical activity to modify the risk of impaired glucose regulation and type 2 diabetes: a study of 16,003 Swedish adults. Diabetes 58: 1411–1418. 19324937 10.2337/db08-1623 PMC2682680

[73] Eichler EE, Flint J, Gibson G, Kong A, Leal SM, et al (2010) Missing heritability and strategies for finding the underlying causes of complex disease. Nat Rev Genet 11: 446–450. 20479774 10.1038/nrg2809 PMC2942068

